# Evaluation of the effect of Retrograde Intrarenal Surgery with Myo-Inositol Oxygenase

**DOI:** 10.12669/pjms.341.14094

**Published:** 2018

**Authors:** Cuma Mertoglu, Aliseydi Bozkurt, Ercüment Keskin, Murat Gunay

**Affiliations:** 1 Cuma Mertoglu, Department of Clinical Biochemistry, Faculty of Medicine, Erzincan University, Erzincan, Turkey; 2 Aliseydi Bozkurt, Department of Urology, Faculty of Medicine, Erzincan University, Erzincan, Turkey; 3 Ercument Keskin, Department of Urology, Faculty of Medicine, Erzincan University, Erzincan, Turkey; 4 Murat Gunay, Department of Clinical Biochemistry, Faculty of Medicine, Erzincan University, Erzincan, Turkey

**Keywords:** Acute kidney injury, Creatinine, Cystatin C, Myo-inositol oxygenase, Retrograde intra-renal surgery.

## Abstract

**Objective:**

To investigate the effect of retrograde intra-renal surgery (RIRS) on kidneys using the myo-inositol oxygenase (MIOX) enzyme. MIOX is a renal tubular-specific novel marker for the early diagnosis of acute kidney injury.

**Methods:**

A total of twenty seven individuals that had undergone RIRS to treat kidney stones were included in the study. Biochemical tests were performed on serum samples collected immediately before RIRS (hour 0) and at the 6th and 24th hours after the surgery.

**Results:**

The creatinine value at hour 6 was lower than the baseline (hour 0) value (p = 0.0305). Cystatin C at hour 6 was lower than the value measured at hour 24 (p = 0.0142). Similarly, MIOX was lower at hour 6 compared to hour 24 (p = 0.0214). MIOX/creatinine at hour 6 was lower than the value calculated at hour 24 (p = 0.0348). The basal values of MIOX and creatinine were found to have a positive correlation (correlation coefficient r = 0.5946, p = 0.0035).

**Conclusions:**

Similar to the serum creatinine, the serum MIOX level provides information about kidney functions. RIRS was confirmed to be a safe procedure for the treatment of acute kidney injury with no negative effects on the kidneys.

## INTRODUCTION

Despite medical developments, acute kidney injury (AKI) remains an important clinical problem with high morbidity and mortality.^1^ Today, the plasma creatinine level is used in AKI diagnosis; however, it is a non-specific and insensitive marker since these levels vary in other conditions causing extrarenal azotemia and do not increase until 50% of kidney functions are lost. In addition, the plasma creatinine level is affected by several other factors such as gender, diet, physical activity and hydration status.^2^

For these reasons, there is an urgent need for a specific and reliable marker for the fast diagnosis of AKI. To date, several enzymes and biomolecules have been investigated, the most promising of which include cystatin C, neutrophil gelatinase–associated lipocalin (NGAL), kidney injury molecule-1 (KIM-1), IL-18, liver fatty-acid binding protein (L-FABP), IL-6, α/π glutathione S-transferase (GST), N-acetyl-β-D-glucosaminidase (NAG), insulin-like growth factor-binding protein 7 (IGFBP7), and tissue inhibitors of metalloproteinases-2 (TIMP-2).^1^,^3^,^4^

In addition to this list, a recent study has demonstrated that the myo-inositol oxygenase (MIOX; EC 1.13.99.1) enzyme has a significant role in AKI diagnosis.^5^ MIOX is the first and only enzyme of myo-inositol catabolism, which is the product of glucose intermediate metabolism, and is predominantly expressed in the kidney.^6^ Cystatin C is a protein produced by all nucleated cells, freely filtered from kidney glomeruli, and almost entirely reabsorbed in the tubules. Due to these characteristics, cystatin C has been suggested as a good marker for the glomerular filtration rate (GFR).^7^ Some of the published research proposed that cystatin C was better than creatinine in relation to an AKI diagnosis. However, other authors have not agreed and considered that creatinine is more powerful a tool for diagnosis.^8^,^9^

The Clinical Guidelines of the European Association of Urology (EAU) recommend retrograde intra-renal surgery (RIRS) together with extracorporeal shockwave lithotripsy (ESWL) for the treatment of kidney stones smaller than 2 cm.^10^ However, RIRS has certain well-known disadvantages such as the long duration of surgery, requirement of additional procedures for larger stones, and high cost.^11^ Since only a limited number of studies have explored the effect of RIRS on kidneys, the current study aimed to investigate the safety of RIRS for kidneys using the MIOX enzyme, a novel renal-specific marker for the diagnosis of AKI.

## METHODS

A total of twenty seven individuals (3 female and 24 male) that had undergone RIRS in our hospital for the treatment of kidney stones were included in this prospective, controlled, and single-centered study. The full approval for the study was received from the ethics committee of Erzincan University (number: 33216249-604.01.02-E.20299, date: 16/05/2016). The study was conducted following the principles for medical research provided by the guidelines of the Declaration of Helsinki and the International Conference on Harmonisation Guideline for Good Clinical Practice. Written informed consent was obtained from all the participants.

All the patients that were to receive surgery were intravenously administered fluid. The fluid replacement started with the administration of anaesthesia and performed for 12 hours with a 50% stable (Ringer's lactate) and 50% isotonic (0.9%) solution at a dose of 1.5 ml/kg / h. After 12 hours, oral fluid replacement was started. The results of the pre-operative tests, including serum biochemistry, urinalysis, and urine culture, ultrasonography, and plain X-ray were obtained. In addition, stone size and location were evaluated together with the anatomical structure undertaking routine computerised tomography.

In cases where the urine culture did not reveal reproduction, RIRS was performed using a flexible uretero-renoscope (3.6 Finside diameter and 7.5 F outside diameter with a 270° angle deflection) (Karl Storz, Endoscopes, Culver City, CA, USA). During the operation, a ureteral access sheath was used to reduce the pressure inside the kidney pelvis. A holmium: YAG laser (General Motor Litho^®^) was used to break down the stones until they were sufficiently small to pass naturally out from the kidneys.

The patients that had an acute infection, a solitary kidney, congenital renal anomalies, urinary tract disorders such as pyelonephritis, diabetes, hypertension, cardiovascular disease, or any other known chronic diseases as well as those that had previously undergone renal surgery or ESWL were excluded from the study. The serum samples were collected from the patients included in the study before RIRS and 6 and 24 hours after the operation. The samples were immediately centrifuged and stored at -80°C until use. The MIOX levels were measured performing ELISA [Cusabio Human inositol oxygenase (MIOX) ELISA Kit] with the results being recorded in pg/ml. The total concentrations of serum creatinine, urea, glucose and cystatin C were analysed using a Beckman Coulter Olympus AU 2700 system.

### Statistical analysis

Data were analyzed using the Statistical Package for Social Sciences Software for Windows version 18.0 (SPSS Inc., Chicago, IL, USA) and descriptive statistics (mean, standard deviation, median, minimum, maximum, number) were generated for the three groups. The normality assumption was checked using the Kolmogorov-Smirnov test. The comparisons of the groups were performed using Wilcoxon Signed-Rank Test. The correlation between AKI markers were tested by Pearson's correlation test. The p-value of <0.05 was considered as statistically significant.

## RESULTS

**Fig. 1:**
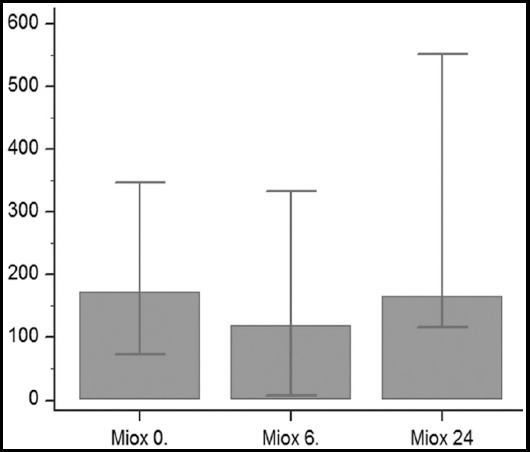
Serum miox levels 95 % CI for median in grafic.

**Fig. 2:**
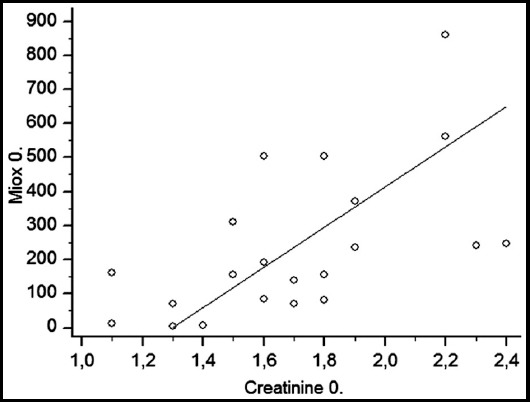
The correlation graph between the MIOX and creatinine values before RIRS (0. hour) (correlation coefficient r = 0.5946, p = 0.0035).

The patient demographic and operative data are shown in Table-I. There was no residual stones (> 4 mm) in the kidney and all patients obstruction was resolved. Creatinine at hour 6 was found to be lower than the baseline value (hour 0) (p = 0.0305). The creatinine value at hour 24 did not differ from the values measured at hours 0 and 6. Cystatin C at hour 6 was lower than the value at hour 24 (p=0.0142). The basal cystatin C value did not significantly change at hours 6 and 24. The MIOX value was lower at hour 6 than at hour 24 (p=0.0214, Fig.1) but the baseline value was no different from the other two measurements. MIOX/creatinine at hour 6 was lower than the value measured at hour 24 (p=0.0348). However, the baseline MIOX/creatinine value did not significantly change at hours 6 and 24. The serum urea, glucose, and MIOX/cystatin C values did not differ between the three measurement times (Tables II and III). A positive correlation was found between the basal values of MIOX and creatinine (correlation coefficient r=0.5946, p=0.0035; Fig.2).

**Table-I:** Demographic and operative data of the patients enrolled into the study.

Parameter	
Age	42±19
Male	24 (88.8 %)
Female	3 (11.2 %)
BMI (kg/m^2^)	29.7±5.6
Previous stone treatment (RIRS)	2 (7.4 %)
ASA score	1.60±0.54
Parenchymal thickness (mm)	16.4±4.8
Mean hounsfield units	880±43.1
Operation time (min)	48±20
Amount of irrigation (cc)	680±140
Laser time (min)	27±16.2
*Grade of hydronephrosis*	
None	0 (0 %)
Mild	6 (22.2 %)
Moderate	13 (48.1 %)
Severe	8 (29.6 %)
*Stent post RIRS*	
Yes	2 (7.5 %)
No	25 (92.5 %)
*Stone location*	
Pelvis	8 (29.6 %)
Calix	7 (25.9 %)
Pelvis + Calix	12 (44.4 %)
Stone Size (mm^2^)	115.2±63.5

Data are presented as mean± SD for continuous variables and as number (percentage) for categorical variables. *Abbreviations:* ASA: American society of anesthesia, BMI: Body mass index, RIRS: Retrograde intra-renal surgery.

**Table-II:** The mean, SD and 95% CI values obtained from the biochemical tests before RIRS (hour 0) and after RIRS (hours 6 and 24).

Parameter(mg/dl)	Mean	SD	95 % CI
Urea 0. h	29	7.7	23.9 - 30
Urea 6. h	25.8	7.1	22.4 – 29.2
Urea 24. h	27	6.6	21.5 – 30.1
Creatinine 0. h	1.68	0.3	1.49 – 1.80
Creatinine 6. h	1.5^a^	0.3	1.4 – 1.6
Creatinine 24. h	1.6	0.3	1.47 – 1.8
Cystatin c 0.h	0.71	0.3	0.54 – 0.88
Cystatin c 6.h	0.61^b^	0.41	0.38 – 0.84
Cystatin c 24.h	0.9	0.34	0.7 - 1.05
Glucose 0. h	89	15	82 - 96
Glucose 6. h	87	33	72 - 103
Glucose 24.h	101	29	88 - 115

a: Different from creatinine hour 0 value, p = 0.0305,

b: Different from cystatin c hour 24 value, p = 0.0142 (SD: Standard deviation, CI: Confidence Interval).

**Table-III:** The median and 95% CI values obtained from the biochemical tests before and after RIRS.

Parameter	Median	95 % CI
Miox 0. h (pg/ml)	157	80 – 248
Miox 6. h (pg/ml)	119^a^	7 – 333
Miox 24. H (pg/ml)	166	115 - 552
Miox 0.h/Cr 0. h	103	50 - 147
Miox 6. h/Cr6. h	35^b^	5 – 170
Miox 24 h/Cr24.h	142	72 – 351
Miox 0.h/ Cys 0.h	282	89 – 553
Miox 6.h/ Cys 6.h	172	29 – 351
Miox 24h/Cys24h	186	122 - 706

a: Different from Miox hour 24 value, p = 0.0214,

b: Different from Miox 24 h/Cr24 h value, p = 0.0348. (Cr: Creatinine, Cys: Cystatin C, CI: Confidence interval, Miox: myo-inositol oxygenase)

## DISCUSSION

One of the most important findings of this study was the demonstration of a moderate positive correlation between serum MIOX and creatinine values for the first time in the literature. This positive correlation demonstrates that the MIOX enzyme can be a marker for renal function, such as creatinine. MIOX is a renal-specific enzyme.^6^ There is a single but significant study that has demonstrated that MIOX increased hours before the elevation of the creatinine level and therefore could be used as a potential early diagnostic marker for AKI.^5^ That study was conducted in two main stages. In the first stage, renal ischemic injury was induced in mice over 30 minutes. The serum MIOX values of the mice were measured at 24 hours and found to be significantly higher than the control group. The second stage involved the evaluation of patients with and without AKI. The MIOX values measured three days before diagnosis were found to increase on average two days before the elevation in the creatinine levels. Using the Western blot technique, it was also shown that MIOX was a renal-specific proximal tubule protein.

In our another study^12^ we also found that MIOX is a significant parameter in the diagnosis of AKI. Similarly, in the present study, we measured the MIOX enzyme to evaluate the effect of RIRS on the kidneys. The lower value of MIOX at hour 6 compared to the baseline value, the lower MIOX/creatinine at hour 6 compared to hours 0 and 24 and the lower cystatin C at hour 6 compared to hours 0 and 24 can be attributed to serum dilation as a result of hydration therapy during the post-operative period. The MIOX value being lower after the surgery (hour 6) similar to the changes in the levels of creatinine and cystatin C indicates that MIOX is as sensitive to dilution as the other two markers. In addition, none of the markers for kidney functions was found to significantly increase after RIRS, which confirms that this procedure is safe for the treatment of AKI with no negative effect on kidney tissue and functions. Similarly, Dede et al.^13^ concluded that RIRS was a safe procedure after finding no significant differences in the urine KIM-1, NGAL, NAG, and L-FABP levels before and after RIRS.

Although several molecules such as NGAL, KIM-1, IL-18, L-FABP, IL-6, α/π GST, NAG, IGFBP7, and TIMP-2 have been reported to have a role in the diagnosis of AKI, none has been put into clinical use due to their specific deficiencies and problems particularly concerning their measurement method.^1^,^3^,^8^ Therefore, there is an ongoing search for a sensitive, specific, reliable, easy-to-measure, and cost-effective marker for the early diagnosis of AKI. Based on the results of this study, MIOX appears to be a promising potential marker.

Cystatin C has been reported to be superior to creatinine in the diagnosis of contrast-induced AKI^14^, AKI after cardiac transplantation^15^, and in patients in the high-risk group.^16^ In contrast, there are also studies that have found that AKI cystatin C is not more effective than creatinine.^9^ In the present study, we observed a decrease in the level of the cystatin C marker at hour 6 due to serum dilation caused by post-operative hydration. In addition, we found that the creatinine and MIOX levels gave similar results in terms of demonstrating the effect of RIRS on the kidneys.

Since MIOX enzyme is an intermediate molecule in the myo-inositol glucose metabolism it catalyses^17^, in this study, the possibility was considered that MIOX would be affected by the glucose levels in the blood. However, the serum glucose levels measured before and after RIRS were not found to be different. Therefore, in the current study, they did not have an effect on the MIOX enzyme.

In a study comparing RIRS with ESWL, the former was reported to cause less pain and to be a more effective treatment.^18^ In another recent meta-analysis, in comparison to ESWL, RIRS was found to offer the patient a greater level of being stone-free, a reduced rate of retreatment, and no increase in the occurrence of complications.^19^

As mentioned above, RIRS is an important treatment option, in particular for kidney stones smaller than 2 cm. This study has contributed to the literature by confirming that RIRS comes into prominence among treatments for kidney injury having no negative effect on the kidneys. But this statement is underlined by present study for kidney function only. On the other hand, it is necessary to be careful to other complications such as sepsis, bleeding, ureter damage.

### Limitations of the study.

The limitations of this study was the lack of a comparison between RIRS and other procedures such as ESWL and PCNL. Therefore, in future studies, the novel marker MIOX should be used to investigate the effect of these two procedures on the kidneys.

## CONCLUSIONS

In conclusion, the positive correlation identified between the serum MIOX and creatinine levels indicate that MIOX also provides information about kidney functions similar to creatinine. In addition, RIRS is a safe treatment procedure with no negative effect on the kidneys.

### Author`s Contribution

**CM:** Conceiving, study designing, data collection, manuscript writing, review and final analysis.

**AB, EK, MG:** Data collection, data interpretation, statistical analysis, discussion writing.
